# Pathophysiology of diabetic kidney disease and autophagy: A review

**DOI:** 10.1097/MD.0000000000033965

**Published:** 2023-07-28

**Authors:** Jiawei Yu, Yan Liu, Hongjie Li, Peirong Zhang

**Affiliations:** a Affiliated Hospital of Weifang Medical University, Weifang Medical University, Weifang, China.

**Keywords:** autophagy, diabetic kidney disease (DKD), hyperglycemia, signaling pathways

## Abstract

Diabetic kidney disease (DKD) is one of the main complications of diabetic microangiopathy. The pathogenesis of DKD is very complex, including autophagy, inflammation, oxidative stress. Although a series of treatment intervention have achieved certain results in the treatment of diabetic nephropathy, still cannot reverse the kidney injury of diabetic nephropathy. The kidney is one of the most important organs of energy metabolism. Renal function is highly dependent on phagocytosis of mitochondria, and aberrant or defective autophagic mechanisms are central to the pathology of many renal diseases. Under high glucose conditions, mitochondrial fragments accumulate in the kidney, suggesting that mitochondrial clearance mechanisms may be attenuated with changes in mitochondrial transformation mechanisms. However, the exact mechanism of mitophagy regulation in DKD has not been elucidated. Recent advances in autophagy have renewed interest in these signaling pathways and molecules in the pathogenesis of DKD. Investigating autophagy and its associated signaling molecules may provide potential unique targets for therapeutic intervention in DKD.

## 1. Introduction

Autophagy, can recognize and degrade damaged macromolecular proteins, organelles, as well as invading pathogens for cellular recycling, which is a biological regulatory process that maintains cellular homeostasis.^[1]^ Increasing attention has been paid to its role in the development of various diseases such as tumors, neurological diseases, diabetes and its complications.^[2]^ The autophagic process mainly consists of 5 stages: initiation, elongation, formation of autophagosomes, fusion and degradation of autolysosomes.^[3,4]^ Biological activity of autophagy is associated with numerous signaling pathways and regulatory factors, which are mainly divided into mTOR-dependent (mammalian target of rapamycin) and mTOR-independent signaling regulation.^[5]^ In recent years, with the development of epigenetic studies, more and more attention has been paid to the regulation of autophagy-related molecule expression.

Studies have shown that autophagy plays a protective role in maintaining glomerular and tubular homeostasis and is important role in human health and disease.^[6]^ In diabetic nephropathy, excessive energy supply inhibits autophagy protection in the kidney, leading to accumulation of damaged proteins and organelles and aggravating the progression of diabetic nephropathy.^[7,8]^ Also, hyperglycemia induces hyperactivation of mTOR complex 1 (mTORc1), which inhibits autophagic activity in podocytes and proximal tubular cells.^[9]^ However, improving autophagic activity in the kidney not only reduces the accumulation of intracellular metabolic damage products and oxidative stress in the kidney, but also improves renal extracellular matrix (ECM) deposition, inflammation and fibrosis, and blocks or delays the development of diabetic kidney disease (DKD).^[10]^ These data suggest that impaired autophagy may be involved in the pathogenesis of DKD, and autophagy activation is promising as a potential therapy for the treatment of DKD.

DKD, as one of the major microvascular complications of diabetes, has become the most important component of hospitalized patients with chronic kidney disease, the pathogenesis has not been fully elucidated, and there are inadequate effective therapeutic agents to control the progression of DKD.^[11]^ Although an increasing number of studies have confirmed that the development of DKD is influenced by factors such as genetics, energy supply, oxidative stress as well as autophagic activity^[12,13]^ and regulation of autophagy-related signaling molecules has also received increasing attention, the role of autophagy in the development of DKD remains inadequately, and more experiments are needed to further improve this field.

In this review, we systematically elaborated the relationship between DKD related pathogenic mechanisms from the beginning of blood glucose and energy metabolism in DKD, and analyze the regulation of DKD pathogenic mechanisms by major autophagy-related signaling molecules, which is of great significance for the treatment of DKD via improvement renal autophagic activity.

## 2. Hyperglycemia and renal cell

Physiologically, it is mainly absorbed into the blood by the villi of intestine and subsequently utilization by cells after food enters the digestive tract. With the increase of blood glucose concentration, pancreatic β-cells secrete insulin and bind to the corresponding receptors on the cell surface, which regulates blood glucose metabolism and maintains the stability of blood glucose and energy.^[14]^ On the one hand, blood glucose entering the cell mainly generates oxygen and energy required for cellular metabolism through mitochondrial electron transport respiratory chain coupled with oxidative phosphorylation via glycolysis and tricarboxylic acid cycle pathways.^[15]^ On the other hand, under hormonal regulation, tissues such as liver, kidney, intestine and muscle use glucose to synthesize glycogen or adipose tissue.^[16]^ Finally, excess circulating glucose is filtered through the glomeruli into the tubular lumen. When the renal glucose threshold is exceeded, blood glucose is excreted in the urine and occurs glycosuria.^[17]^

In general, the cell surface insulin-independent glucose transporter 1 (GLUT1) is involved in regulating intracellular and extracellular glucose transport, but different tissue and cell types have different ability to regulate intracellular and extracellular glucose.^[18]^ Nurit Kaiser et al found that GLUT1 protein was more abundant in smooth muscle cells compared with endothelial cells, and reduced by approximately 50% in smooth muscle cells when the glucose concentration of the medium was increased from 1.2 to 22 mmol/L for 24 hours.^[19]^ In addition, aldose reductase is a catalytic enzyme widely distributed in many tissues of the human body involved in the formation of glycation products.^[20]^ It has the highest immunological activity in the medullary part of the kidney, but its expression is very low in tissues such as the stomach, intestine, liver, spleen, and lung.^[21]^ Therefore, kidney, heart and other major diabetic target organs damage may be closely related to specific molecular biological characteristics in these tissues. However, understanding what pathophysiological changes occur in the kidney of diabetic hyperglycemia is particularly necessary to understand the pathological process of DKD.

## 3. Hyperglycemic metabolic stress and renal injury in DKD

In addition to oxidative stress, renal cell injury in DKD mainly includes 4 pathogenic mechanisms: activation of polyol metabolic bypass, generation of Advanced Glycation End products and activation of their receptors (AGEs-RAGEs), activation of protein kinase C (PKC), and overactivation of hexosamine bypass.^[22]^ However, clinical studies have found that blocking either of the above mechanisms of action alone is not effective in blocking the progression of DM (diabetes mellitus) microangiopathy . The intermediate products of the glycolytic pathway are closely related to the formation of the above 4 pathogenesis of DKD, while a large amount of reduced NADH/FADH2 products via the tricarboxylic acid cycle enters the mitochondrial electron transport respiratory chain generate superoxide products is considered to be a common factor in the accelerated progression of the above pathomechanism.^[24,25]^ Therefore, it will help to understand the overall mechanism of DKD by investigating how hyperglycemia leads to increased intracellular oxidative stress and the internal connection to the above 4 pathogenic mechanisms.

### 3.1. Hyperglycemia leads to increased oxidative stress

Physiologically, glucose enters tissue cells to successively generate ATP via the glycolytic pathway, the tricarboxylic acid cycle, and mitochondrial electron transport respiratory chain coupled with oxidative phosphorylation to provide the energy required for survival and metabolism of tissue cells.^[26]^ However, under hyperglycemic conditions, a large amount of glucose enters the tricarboxylic acid cycle to generate a large amount of NADH and FADH2, which increase the voltage gradient on both sides of the mitochondrial inner membrane, resulting in electrons carried by CoQ are unable to be efficiently transmitted to complex III, not to oxygen molecules, leading to superoxide generation,^[27]^ which is degraded to hydrogen peroxide (H2O2) by superoxide dismutase from mitochondria, generates water and oxygen atoms. However, under the DM, hyperglycemia activates the polyol bypass, resulting in the consumption of large amounts of NADPH, production of superoxide dismutase (SOD) decreased, which relies on NADPH anti-oxygen free radicals and eventually intracellular reaction oxygen species (ROS) and oxidative stress increased.^[28]^

### 3.2. Hyperglycemia and oxidative stress contribute to the development of DKD

Multiple pathogenic mechanisms of DKD originate from intermediates in glucose glycolysis, and oxidative stress resulting from hyperglycemia contributes to the development of the above 4 pathogenic mechanisms of DKD. Physiologically, glyceraldehyde 3-phosphate generates glycerate 1,3 diphosphate under the action of its dehydrogenase, following continues glycolysis and enters the tricarboxylic acid cycle, but hyperglycemia inhibits the activity of glyceraldehyde 3-phosphate dehydrogenase, resulting in accumulation of large amounts of prophase glycolytic products, which leads to polyol bypass activation.^[28]^ Glucose undergoes the action of aldose phosphatase to generate sorbitol, which consumes a large amount of NADPH, which reduces the synthesis of reduced glutathione that scavenges superoxide and causes an intracellular oxidation-antioxidant imbalance, resulting in increased intracellular oxidative pressure.^[29]^ A pervious study suggest that mitochondrial DNA damage is likely to occur with increased superoxide in mitochondria and resulting in activating poly (ADP-ribose) polymerase required for DNA repair, although the activation of this enzyme has a certain positive effect on mitochondrial DNA repair, it also inhibits glyceraldehyde 3-phosphate dehydrogenase activity like hyperglycemia, reduces the reaction rate of the glycolytic pathway, and aggravates the development of the pathogenic mechanisms of DKD.^[30]^ However, another study showed that hyperglycemia did not increase in production of ROS when the voltage gradient on the mitochondrial membrane was disrupted by uncoupling protein 1 (UCP-1) or generated oxygen radicals were degraded by manganese superoxide dismutase (MnSOD). Thus, oxidative stress resulting from hyperglycemia is thought to be an upstream signaling pathway common to pathogenic mechanisms.

## 4. Oxidative stress and the 4 pathogenic mechanisms in DKD

Oxidative stress and the above 4 pathogenic mechanisms lead to renal injury mainly via 2 ways. The first is through affecting multiple balance systems, such as oxidation-antioxidant balance, protein homeostasis and mitochondrial quality control, the second is through regulating the gene transcription and translation processes of intracellular DKD-related pathogenic molecules, such as PKC activation and hexosamine pathway hyperactivation.

### 4.1. Oxidation-antioxidant balance

Hyperglycemia induced glucose metabolism disorders are the main cause of massive generation of superoxide and increased oxidative stress in the kidney. In order to reduce the damage of oxidative stress to cells, the body initiate antioxidant defense systems to scavenge free radicals.^[31]^ Under the regulation of NADPH/NADP^+^, glutataione (GSH) and cysteine (Cys) undergo the interconversion of oxidative and reduced states, so they can be continuously activated and inactivated according to the desired state, so reduced glutathione continuously acts as an electron donor to reduce H2O2 to water and maintaining intracellular oxidative balance.^[32,33]^ However, due to the inhibitory effect of hyperglycemia and oxidative stress on glyceraldehyde 3-phosphate dehydrogenase in glycolysis, the polyol bypass pathway is activated, which not only consumes a large amount of NADPH, but also reduces the production of reduced glutathione, fails to remove a large amount of superoxide in time, which relies on NADPH, resulting in aggravating intracellular oxidative damage.^[34]^

### 4.2. Proteostasis

Unlike the reversible redox reactions mentioned above, protein carbonylation is a non-enzymatic protein oxidation, which is regarded as a marker of severe chronic oxidative damage due to causing irreversible damage to proteins.^[35,36]^ In order to maintain intracellular proteostasis, the cell itself initiates a series of mechanisms to maintain proteostasis, and the proteasome system is the most important intracellular protein degradation mechanism (mainly degrades small proteins, while large folded proteins and organelles require autophagic system degradation). It catalyzes the degradation of proteins that are no longer needed, damaged, or even weakened by the cell itself in an ATP-dependent manner by recognizing and combining hydrophobic structures exposed after protein damage.^[37,38]^ In addition, to prevent oxidatively damaged proteins from accumulating during mild oxidative stress, chaperone heat shock proteins refold damaged proteins by recognizing and binding exposed hydrophobic protein sequences, or leave unfolded proteins in a dissolved state to avoid forming multimers with other damaged proteins.^[39,40]^

### 4.3. Mitochondrial quality control is unsustainable

Mitochondria is not only the starting point of oxidative stress, but also the main target organelle of oxidative damage and the repair, regeneration and autophagic degradation of mitochondria to maintain the quality control of mitochondria.^[40,41]^ There is a study about mitochondrial quality control in vitro showed that when mitochondria are damaged, PINK1 exposure on the outer membrane surface increases, where it can activate the ubiquitin ligase of parkin, ubiquitinates the mitochondrial membrane surface protein (Lys63), and binds to the adaptor protein P62 of autophagy, meanwhile it binds to ATG8 on the autophagic vacuole membrane through the microtubule associated protein light chain 3 interacting region (LIR), then promotes recruitment of ubiquitin binding mitophagy receptors to promote capture by the autophagosome.^[42]^ Newly formed autophagic vacuoles firstly associate with acidified endosomes to form autophagosomes and then fuse with lysosomes to form autolysosomes.^[43]^ Thus, damaged organelles or biomacromolecules are degradated to nucleotide fragments and fatty acids, which is not only maintains intracellular proteostasis, but also releases degradation products into the cytoplasm to provide raw materials for new biosynthesis. In the DKD state, renal autophagy activity is inhibited, resulting in increased accumulation of injured mitochondria, which further aggravates intracellular oxidative stress and renal structural and functional impairment.^[44]^ In addition, it has been reported that increased PINK1 protein is also able to activate peroxisome proliferator-activator receptor gamma coactivator 1α (PGC-1α) of the main regenerative regulatory molecule with promoting autophagy, following activates downstream nuclear respiratory factor (NRF) and mitochondrial transcription factor A (TFAM),^[45]^ to synthesizes new mitochondria to maintain the quantity and quality stability of mitochondria. Thereby, PGC-1α expression in the kidney is significantly downregulated in the DKD state, resulting in reduced mitochondrial production, and reduced synthesis of important proteins such as ATPase in injured mitochondria, insufficient ATP supply as well as caused impaired energy supply in the DKD, which are also important factors which aggravates structural changes and functional impairment in the kidney.^[46]^

### 4.4. Activation of PKC signaling

PKC activation and increased oxidative stress can regulate the expression of a variety of DKD related pathogenic genes, such as downregulation the expression of nitric oxide synthase (NOS) of the vascular endothelial relaxing factor and upregulation the expression of endothelin 1 of the vascular endothelial contractile factor to reduce renal blood flow and improve renal hemodynamics.^[47]^ It can also promote the expression of collagen fibers, proinflammatory factors and accelerate renal fibrosis through transcription factor β, plasminogen activator inhibitor 1 (PAI-1) and nuclear transcription factor-κB (NF-κB). In diabetic Klotho deficiency mice, the expression of PKC-α and p66SHC was upregulated and subsequently promoted the generation of ROS, resulting in podocyte injury and aggravating proteinuria levels.^[48]^ So, PKCα/p66SHC may play a role in mediated podocyte injury in diabetic nephropathy.

### 4.5. Hyperactivation of the hexosamine pathway

Hyperactivation of the hexosamine pathway culminates in the formation of uridine diphosphate and N-acetylglucosamine, the latter inhibits the phosphorylation of Akt/eNOS and HSP72 through kinase-like effects of serine/threonine phosphorylation, lead to increase the expression of transforming growth factor-β (TGF-β) and PAI-1, and promote oxidative stress and fibrosis in the DKD.^[49]^

## 5. Inflammation and DKD

Increasing evidence from clinical and experimental studies suggested that inflammation play a critical role in the development and progression of DKD, such as macrophages, and inflammatory cytokines, nuclear factor κB (NF-κB), Janus kinase/signal transducer and activator of transcription (JAK/STAT) pathways.^[50]^ It has been found that the mononuclear phagocyte system is activated in DM, releasing cytokines and recruiting, ultimately leading to inflammation-related structural changes.^[51]^ In addition, mast cells are capable of infiltrating the tubulointerstitium and releasing inflammatory mediators and proteolytic enzymes, which may be associated with decreased glomerular filtration rate (eGFR).^[51]^

Cytokines are a group of polypeptide signaling molecules. In the kidney, different kidney cells can synthesize inflammatory cytokines, such as IL-1, IL-6, IL-18, and TNF-α, all of which are different extent involved in the pathogenesis of DKD.^[52]^ In models of DN, renal expression of IL-1 increases is related to subsequent expression of chemotactic factors.^[53]^ Another study also showed that IL-1 directly increases vascular endothelial cell permeability.^[54]^ Nosadini et al reported that IL-6 can promote neutrophil infiltration into tubulointerstitium, affecting ECM dynamics, and promoting overall renal hypertrophy, glomerular basement membrane thickening, which is associated with albuminuria.^[55]^ TNF-α is capable of inducing and differentiating inflammatory cells, cytotoxicity to renal cells, activating apoptosis, altering glomerular hemodynamics, increasing vascular endothelial permeability, and oxidative stress.^[56]^ It has been shown that TNF-α mRNA expression levels are elevated in glomerular and tubular cells during the early stages of diabetes. In db/db mice, caspase inhibitors reduced renal CASP1, IL-1β, IL-18, and NLRP3 inflammasome activation and ameliorated albuminuria and renal ECM accumulation.^[57]^

JAK/STAT is an intracellular cytokine related signaling pathway that plays an important role in mediating paracrine stimulation and nuclear receptors.^[58]^ Cytokines can regulate cell activation, proliferation, recruitment, migration, and differentiation by activating this fundamental mechanism. It has already been reported that JAK/STAT was upregulated in glomerular from patients with early DKD and tubulointerstitial expression of various JAK and STAT isoforms increases with disease progression and is inversely correlated with eGFR.^[59]^ NF-kB is a key transcription factor activated by cytokines and oxygen radicals during renal inflammation in diabetes and is activated through JAKs/STATs, leading to the structural alterations and functional abnormalities in DKD.^[60]^ In renal cells, NF-kB is rapidly activated by multiple stimuli, including hyperglycemia, AGEs.^[61]^

The kidney has been found to be the earliest organ of AGEs injury in diabetes, meanwhile is also the main organ of AGEs filtration clearance.^[62]^ Hyperglycemia and oxidative stress increase AGEs and RAGEs production, resulting in a rapidly increase in oxidative stress and NF-κB expression in endothelial cells, which not only promotes adhesion molecule transcription, but also promotes glomerular epithelial cell differentiation into interstitial cells, reduces NOS activity, and accelerates renal vasoconstrictor dysfunction and vascular sclerosis.^[63]^ In addition, it has also been suggested that AGE diffusing out of cells and its precursors damage albumin in the circulation and activate the transcription and secretion of systemic inflammatory mediators.^[64,65]^ Although no significant benefit has been found in DKD-III disease patients with anti-AGE agents in clinical trials,^[66]^ first-line drugs such as metformin for the treatment of DM/DKD, were shown to be able to reduce tubulointerstitial injury by activating AMP-activated protein kinase (AMPK), blocking the AGE-RAGE axis, and reducing the production of ROS and matrix metalloproteinase 2.^[67–69]^

Of course, these modes of action are not isolated, and they tend to synergize to promote the progression of DKD. The key to the generation and aggravation of hyperglycemia and oxidative stress is to lead to metabolic abnormalities in vivo, while autophagy, as a major catabolic pathway, is involved in the degradation and recycling of macromolecules and damaged organelles to maintain intracellular homeostasis, leading to hypothesize that autophagy plays an important role in the development and progression of diabetic nephropathy and is a promising treatment option.

## 6. Regulation of pathogenic mechanism of DM/DKD via autophagy associated molecules

mTOR, AMPK and NAD-dependent deacetylase sirtuin-1 (SIRT1) are an important regulator regulating mitochondrial function and autophagic activity.^[70–73]^ It has been found that these signaling molecules are involved in multiple links in the development of type 2 diabetes as well as diabetic nephropathy.^[71]^

### 6.1. Mammalian target of rapamycin

mTOR belongs to the PI3K-related protein kinase family and is a serine/threonine protein kinase, which consists of mTORC1 and mTORC2, and its activity correlates with intracellular nutrient levels and redox status.^[74,75]^ The core complex of mTORC1 consists of mTOR, regulatory protein with mTOR, and mLST8 protein (mammalian lethal with Sec13 protein 8),^[76,77]^ which regulate cell growth mainly by promoting translation, ribosome biogenesis to inhibit intracellular autophagic activity.^[78]^ mTOR, as a major regulator of autophagy, is regulated by multiple upstream signaling pathways to directly or indirectly regulate related gene expression or protein modification.^[79,80]^

It has been found that a high-fat diet can overactivated mTORC1 and inhibit phosphorylation at the serine site of insulin receptor substrate 1 (IRS-1 Ser312 and Ser636 sites), thereby increasing insulin resistance in vivo,^[81]^ causing inhibition of glucose uptake and glycogen synthesis.^[82]^ Sakaguchi et al found that mTOR activation was induced by hyperglycemia was closely associated with proliferation and apoptosis of tubular cells in diabetic nephropathy, while tubular injury was ameliorated after knocking down the mTOR gene in proximal tubular cells.^[83,84]^ Another study also showed that rapamycin, as a mTOR inhibitor, was not only able to increase blood glucose uptake by 17% and improve insulin resistance, but also significantly prolong the survival time of species.^[77]^ In addition, rapamycin is also able to reduce glomerular injury via multiple ways, such as improving glomerular basement membrane thickness, tubular epithelial transdifferentiation to mesenchymal cells, macrophage recruitment, reducing proteinuria, as well as improving renal autophagic activity.^[85]^[Bibr R23]

### 6.2. SIRT 1

SIRT 1 is a member of the Sirtuins family of NAD-dependent deacetylases in mammals, and it is able to deacetylate a variety of substrate proteins in the presence of nicotinamide adenine dinucleotide (NAD+) and is involved in the repair process of multiple cell injuries such as anti-oxidative stress, autophagic activity and anti-aging, thereby improving multiple pathogenic mechanisms of DM/DKD.^[86,87]^ Watanabe et al reported that inhibition of SIRT1 leads to activation of the mTOR pathway and decreases AMPK activation,^[88]^ activate autophagy-related proteins such as Atg5 by deacetylation, which promotes autophagic activity.^[89]^ In addition, sirt1 is also able to promote the synthesis of autophagosomes and lysosomes through activating the PINK1/parkin pathway, enhancing intracellular autophagy levels.^[89]^ Therefore, sirt1 may promote autophagy levels in different cells by regulating multiple links associated with autophagy.

The expression level of SIRT1 protein is closely related to the development of diabetic nephropathy as well as hyperglycemic status. In STZ-induced and db/db diabetic kidneys, Sirt1 expression significantly decreases, which maybe due to Sirt1 activity is strictly regulated by intracellular NAD + concentrations, which are likely to decrease in diabetic organs.^[87,90]^ It has been found that resveratrol, a SIRT1 agonist, can enhance the expression of SIRT1, not only improve blood glucose homeostasis and insulin resistance, promote mitochondrial regeneration, but also reduce mesangial cell proliferation, improve renal autophagy activity and reduce inflammation as well as reduce oxidative stress, play a role in the treatment of diabetic nephropathy.^[91,92]^ In addition, in response to fasting, SIRT1 not only enhances gluconeogenesis and inhibits glycolysis by interacting with PGC-1α, FOXO 1, CRTC2,^[93]^ but also maintains energy supply and blood glucose stability through the regulation of PGC-1α and peroxisome proliferator activated receptor-α (PPAR-α), which promotes fatty acid oxidation, hepatic glucose output and gluconeogenesis.^[91]^

### 6.3. AMPK

AMPK is a member of serine/threonine protein kinase family, which consists of 3 subunits, a catalytic subunit α and 2 regulatory subunits β and γ and it is biologically active after the threonine 172 of its α subunit is phosphorylated,^[94]^ is highly expressed in various kidney cells.^[95]^ In diabetes experimental models, the expression of AMPK was downregulated and phosphorylation levels of AMPK were also significantly reduced in glomerular epithelial cells,^[96]^ while improving expression levels of AMPK has been found to restore energy homeostasis and improve insulin resistance.^[97]^ In addition, AMPK also plays a role in the treatment of DKD by improving renal filtration barrier injury and autophagic activity, reducing ECM deposition and renal fibrosis.^[98,99]^

AMPK is also a major sensor protein of energy status in eukaryotic cells and is able to inhibit mTOR and reduce protein synthesis according to changes in intracellular energy, thereby preferentially ensuring the energy supply of cells.^[100]^ When the utilization rate of blood glucose decreases, ATP synthesis decreases, while AMP increases, increased AMP binds and activates AMPK, following serine at positions 1345 of tumor suppressor (TSC2) is phosphorylated by activated AMPK and has GTPase activity, leading to GTP hydrolyze to GDP, which weakens the binding of GTP-dependent Rheb to mTOR molecules, exerting the effect of inhibiting mTOR, thereby enhancing the autophagic activity of cells.^[101]^ Another study also showed that GSK3 phosphorylated TSC2 only when TSC2 had been previously phosphorylated by AMPK, whereas mutation of the AMPK site S1345A in TSC2 completely eliminated GSK3 phosphorylation, demonstrating that AMPK-priming phosphorylation was required for subsequent GSK3 phosphorylation, eventually inhibits mTORC1 expression.^[102]^ Recently, it has been found that AMPK is able to directly phosphorylate the raptor subunit of mTORC1 as predicted by tandem mass spectrometry and TSC2 knockdown analysis, thereby directly exerting an inhibitory effect on mTOR.^[103]^

## 7. Conclusion

In summary, there are also complex interaction networks between these autophagy-related signaling molecules, which are jointly involved in the regulation of multiple pathogenic factors in diabetic nephropathy. Current ample research evidence supports that multiple DM vascular complications, including DKD, can be ameliorated by modulating these signaling molecules. In diabetic conditions, altered expression of these stress-stimulated autophagy molecules may be beneficial in improving organelle dysfunction and subsequent worsening of diabetic nephropathy. Therefore, we provide a systematic review of the therapeutic effects of autophagy on diabetic nephropathy to help future research in this area.

## Author contributions

**Writing – original draft:** Peirong Zhang, Jiawei Yu, Yan Liu, Hongjie Li.

**Writing – review & editing:** Peirong Zhang, Jiawei Yu.
